# Maintenance therapy with subcutaneous immunoglobulin in a patient with immune‐mediated neuropathic postural tachycardia syndrome

**DOI:** 10.1016/j.jtauto.2021.100112

**Published:** 2021-08-14

**Authors:** Kalliopi Pitarokoili, Andrea Maier, Elena C. de Moya Rubio, Katrin Hahn, Gerd Wallukat, Diamantis Athanasopoulos, Thomas Grüter, Jeremias Motte, Anna Lena Fisse, Ralf Gold

**Affiliations:** a Department of Neurology, St. Josef-Hospital, Ruhr-University Bochum, Bochum, Germany; b Immunmediated Neuropathies Biobank (INHIBIT), Ruhr-University Bochum, Bochum, Germany; c Department of Neurology, University Clinic Aachen, Germany; d POTS und andere Dysautonomien e.V. (German Patient Organization, NGO), Bochum, Germany; e Department of Neurology and Outpatient Clinic, Charité – University Berlin, Berlin, Germany; f Berlin Cures GmbH, Berlin, Germany

**Keywords:** Small fiber neuropathy, Autoimmunity, Neuropathic postural tachycardia syndrome, Subcutaneous immunoglobulin

## Abstract

**Aims:**

We describe the disease course of a 35-year-old female with an autoimmune mediated neuropathic postural tachycardia syndrome (PoTS), who responded to immunoglobulin therapy and stabilized on maintenance therapy with subcutaneous immunoglobulin (SCIg).

**Methods:**

We provide longitudinal data of clinical scores, tilt-table results and antibody titers.

**Results:**

Initial treatment with intravenous immunoglobulin caused infusion-related side-effects whereas SCIg was well tolerated and improved clinical symptoms and quality of life. Clinical improvement correlated with the reduction of serum antibody titers 22 months after first infusion.

**Conclusions:**

These findings suggest that autoimmune-mediated neuropathic PoTS can be treated sufficiently with IVIg whereas SCIg minimizes side-effects.

## Highlights

• A 35-year-old female with an autoimmune-mediated neuropathic postural tachycardia syndrome (PoTS) responded to immunoglobulin therapy and subcutaneous immunoglobulin (SCIg).
• Treatment with IVIg caused infusion-related side-effects SCIg was well tolerated and improved clinical symptoms and quality of life.
• Clinical improvement correlated with the reduction of serum antibody titers 22 months after first infusion.

## 1 Introduction

Postural tachycardia syndrome (PoTS) is defined as a heart rate increase of more than 30 bpm and/or heart rate of more than 120 bpm within 10 minutes of standing accompanied by symptoms of autonomic dysregulation (orthostatic dizziness, palpitations, presyncopes or syncopes, fatigue) over a period of at least 6 months [1,2]. Identifying and treating the specific cause of PoTS in each patient is important. Different causes and subtypes of PoTS are discussed and still under investigation. Besides hypovolemia, anemia, neurodegenerative aspects and connective tissue disorders, autoimmunity seems to play an important role in the pathogenesis of PoTS in some patients [[3], [4], [5], [6], [7]]. Moreover, small fiber neuropathy (SFN, neuropathic PoTS) could be detected in around 50 % of patients with PoTS [[8], [9], [10]]. Small fiber neuropathy preferentially affects unmyelinated C-fibers, thinly myelinated A-δ somatosensory axons and sympathetic and parasympathetic neurons. Patients typically present with somatosensory complaints such as neuropathic pain but can also present with autonomic involvement [[11], [12], [13]].

However, even after excluding underlying common (such as diabetes mellitus and HIV) but also rare causes that may be potentially treatable (Fabry disease, Sjögren syndrome, celiac disease) [11], the proportion of patients with idiopathic SFN ranges from 24% up to 93% [[13], [14], [15], [16]]. On the other hand, up to 57 % of PoTS patients are suspected to have a SFN (neuropathic PoTS) [[8], [9], [10]].

Both PoTS and SFN can be considered autoimmune due to the presence of autoimmune comorbidities, autoantibodies and inflammatory changes in the nerves [[3], [4], [5], [6], [7]]. Consequently, a variety of clinical studies have already reported or currently investigate the immunomodulatory effect of intravenous immunoglobulin (IVIg) for the treatment of autoimmune SFN [14,[16], [17], [18], [19], [20], [21]].

The term ‘autoimmune neurosensory dysautonomia’ in combination with possible mechanisms (mostly anti-G protein coupled receptors autoantibodies) has been proposed in order to describe these seemingly unrelated symptoms [5]. Indeed, a variety of antibodies against adrenergic α - and β -, angiotensin II type 1, muscarinic 1–5 and nociceptin-like receptors have been detected in series of PoTS patients but not in control sera [[22], [23], [24]].

Here, we report the case of a female with an autoimmune-mediated neuropathic PoTS, initial improvement of symptoms with IVIg but an impressive low side-effect profile with SCIg. Thus, a considerable increase of quality of life after administration of subcutaneous immunoglobulins (SCIg) could be attained.

## 2 Case description and results

**Fig. 1:**
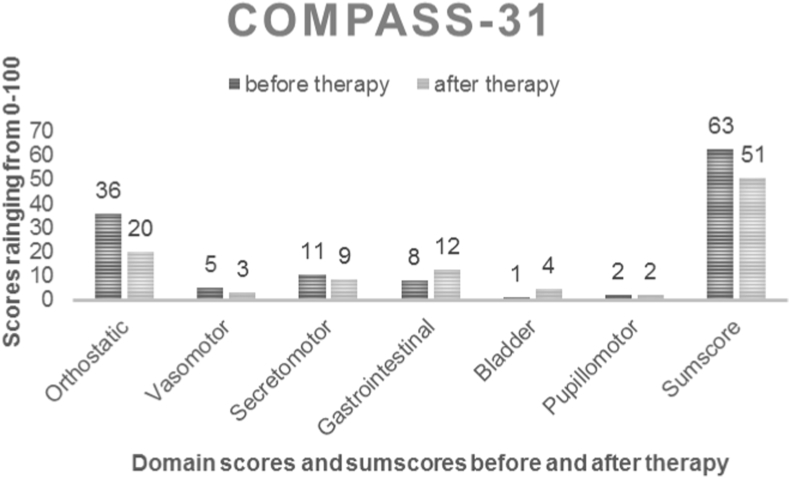
COMPASS-31 final domain scores (orthostatic, vasomotor, secretomotor, gastrointestinal, bladder, pupillomotor) and the sum score before and after therapy. There was an improvement in orthostatic, vasomotor and secretomotor symptoms and in the sum score.

A 35-year-old Caucasian female experienced for the first-time after a severe upper respiratory infection progressive, symptoms of orthostatic dysregulation including orthostatic headaches, near fainting and fainting, cognitive impairment, restlessness and fatigue after standing, prolonged sitting or after short walks (Fig. 1). The infection occurred 2 weeks after a routine pneumococcal vaccination due to a Marfan Syndrome. Further symptoms of autonomic neuropathy included occipital neuralgia, sound and smell hypersensitivity, gastrointestinal problems (reflux, nausea, postprandial bloating and pain, diarrhoea, which evolved to obstipation in a later stage), exercise intolerance, sleep and temperature dysregulation, dry mouth, eyes and facial skin, hyperhidrosis, blood pooling in the lower extremities and also signs of small fiber neuropathy as burning feet and hands. A variety of physicians (neurologists and cardiologists) failed to diagnose in the first 18 months the progressive disability due to autonomic symptoms [25], an iron deficiency was diagnosed and treated, but symptoms did not improve.

PoTS was diagnosed in a specialized outpatient university clinic for disorders of the autonomic nervous system. The tilt table test demonstrated the presence of PoTS with a heart rate increase by 60 bpm up to a total of 137 bpm accompanied by symptoms within 10 minutes standing, without orthostatic hypotension.

Standing serum norepinephrine was elevated to 902 ng/l (>600 pg/mL) and a hyperadrenergic tumour was excluded.

The first quantitative sensory testing (QST) showed signs of a dysfunction of the A-δ and C-fibers with central sensitization and a non-length dependent pattern. A skin biopsy from the distal lower leg 10mm above the malleolus lateralis confirmed a severe SFN with an intraepithelial nerve fiber density (IENFD) of 0,2/mm [28]. Antibodies against adrenergic β1 and β2, muscarinic M2 and M4 and against nociceptin-like receptors were positive in three different laboratories (ELISA and life-based immunoassay on cardiomyocytes – Table 1)*.*

**Table 1:** Antibody activity before and after immunoglobulin (Ig) treatment. The cut off value is 1.8 LU (laboratory units) for healthy persons, these values were established in Berlin Cures GmbH, Berlin.

Antibodies against	Before Ig	After Ig
β2 adrenergic receptors (LU)	6	2.3
M2 muscarinic receptors (LU)	5.2	3
Nociceptin-like receptors (LU)	4.5	3.7

PoTS alleviating medications (clonidine 675 μg/day, β-blockers such as nebivolol 5mg/day or propranolol 80mg/day or bisoprolol 2,5mg/day, etilefrinhydrochloride 10mg/day, midodrine 7,5mg/day, higher doses not tolerated) and further treatment like compression stockings, high salt and liquids diet, adapted PoTS exercise program failed to provide sufficient relief. The patient is an interpreter, smokes 5 cigarettes a day for the last 10 years and a social drinker. Regarding family history the father was also diagnosed with Marfan syndrome and the grandfather with Parkinson's disease. A deep vein thrombosis with secondary lung embolism was diagnosed 18 months after first PoTS manifestation.

Known genetic mutations for SFN and dominant ones for classical and vascular Ehlers-Danlos were excluded. The full diagnostic criteria for a mast cell activation syndrome [26] were not fulfilled, however gastrointestinal and cutaneous symptoms improved with antihistamines and worsened by adrenalin rushes. Lactose intolerance, small intestinal bacterial and intestinal methanogen overgrowth were diagnosed later in the course of the disease [27].

18 months after the onset of symptoms, an autoimmune mediated, neuropathic POTS refractory to symptomatic treatment was diagnosed. A dose of 2 g/kg body weight IVIg over 5 days was administered 33 months after the first manifestation of symptoms. However, this infusion and the following ones (1 g/kg IVIg) were poorly tolerated and had to be performed at a rate of 2–3 g/h in an inpatient clinic as symptoms of orthostatic dysregulation and other side effects as flu-like symptoms, headaches and nausea were exacerbated during the infusion. Nonetheless, 3 weeks after the first infusion the patient reported an improvement of the orthostatic symptoms. After a total of 11 cycles of 1 g/kg IVIg every 4–8 weeks over 3 days a gradual improvement of fatigue, dizziness and near fainting was reported (Fig. 1). Subsequently, headache during and after IVIg, the extreme fatigue and worsen sleep dysregulation after the infusion led to the switch to a maintenance therapy with SCIg (0,25 g/kg every week). After this treatment adjustment a further impressive stabilization of the cardiovascular, neurological, and cognitive symptoms followed for the next 6 months without treatment-related fluctuations or side-effects. Clonidine was reduced from 675 to 525 μg/day, midodrine was not needed any more.

**Fig. 2:**
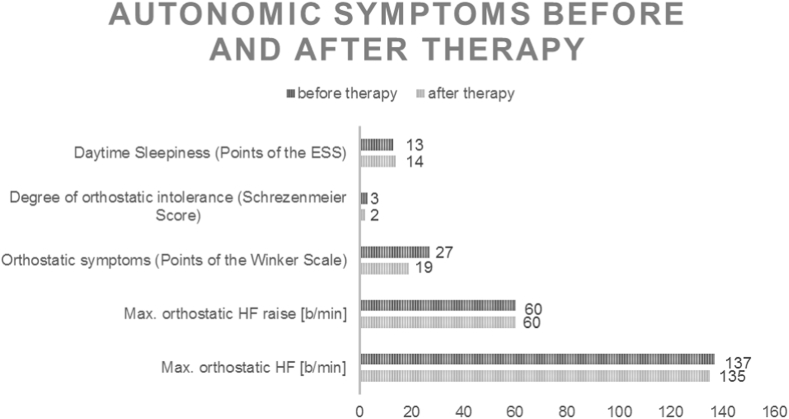
Whereas heart rate parameters in tilt table examination and daytime sleepiness (ESS = Epworth sleepiness Scale) did not worsen over time, orthostatic symptoms (sum score of points of the Winker Scale) improved by therapy. Orthostatic intolerance improved from 3 points before therapy (orthostatic symptoms occur in most occasions and orthostatic stress regularly produces symptoms, impairment of daily activities, standing time about 1 min) to 2 points (orthostatic symptoms occur frequently, at least once a week and orthostatic stress usually produces symptoms, standing time about 5 minutes). HF=Heart rate.

Whereas heart rate parameters in tilt table examination and daytime sleepiness did not worsen over time, orthostatic symptoms improved by therapy (Fig. 2). The patient had less and later PoTS symptoms and no more syncopes during standing. There was an improvement in orthostatic, vasomotor and secretomotor symptoms and in the sum score of the Composite Autonomic Symptom Score (COMPASS-31) questionnaire [29].

**Fig. 3:**
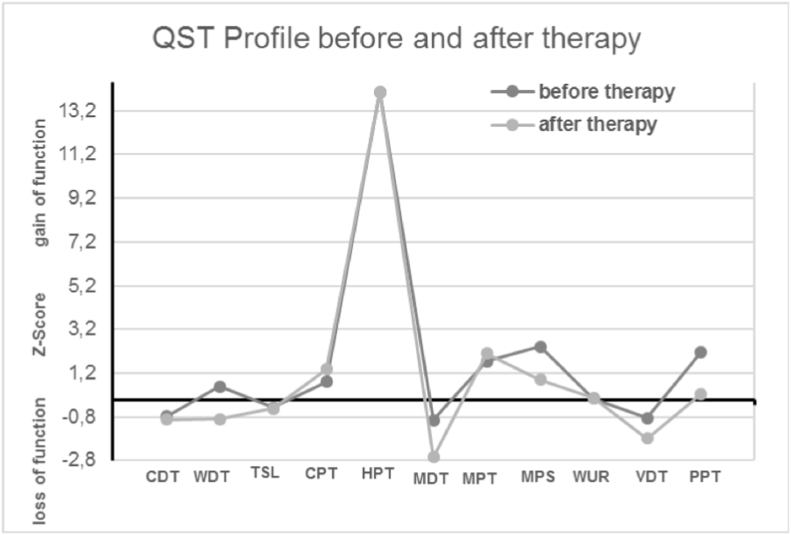
Quantitative sensory testing before and after treatment: the z-profile in QST at the left side shows a central sensitization before treatment (gain of function in PPT and MPS, 2 paradoxical heat sensations), which improves under therapy. Hyperalgesia (gain of function in MPT) and hypesthesia (loss of function in MDT and CDT) developed over time. CDT = cold detection threshold; WDT = warm detection threshold; TSL = thermal sensory limen; CPT = cold pain threshold; HPT = heat pain threshold; MDT = mechanical detection threshold; MPT = mechanical pain threshold; MPS = mechanical pain sensitivity; WUR = wind-up ratio; VDT = vibration detection threshold; PPT = pressure pain threshold; DMA = dynamic mechanical allodynia; PHS = paradoxical heat sensation.

Central sensitization before treatment improved under therapy (Fig. 3). 24h electrocardiogram (heart rate of 55–130 bpm, no fainting) and 24h blood pressure measurement confirmed the clinical findings. Antibodies titers against adrenergic β2, –, muscarinic M2 and nociceptin-like receptors were reduced after treatment (Table 1).

## 3 Discussion

This case demonstrates a treatment response on immunomodulating therapy with IVIg followed by a maintenance therapy with SCIg in a case of neuropathic PoTS with relevant infusion-related side-effects during IVIg treatment.

PoTS and SFN are underestimated disorders both regarding its prevalence as well as its treatment options. Compared to the emerging research on large-fiber neuropathies, where immune-mediated polyneuropathies have been better characterised, little is known on the pathophysiology of SFN [30,31]. Both SFN and PoTS lead, however, to a comparable impairment of the quality of life [32,33]. Treatment options remain symptomatic although devastating and progressive symptoms such as autonomic dysfunction and pain are its main characteristics reducing quality of life and physical performance.

Most case reports on SFN depict this diagnostic and therapeutic gap and describe the odyssey of patients until definite diagnosis, the inadequate stabilization with symptomatic treatment and the lack of epidemiological studies to evaluate the dimension of the problem. The same odyssey is reported for patients with PoTS, since the differential diagnoses of this autonomic dysfunction comprise a wide variety of concomitant aetiologies (hypovolemic, hyperadrenergic, autoimmune, neuropathic and/or cholinergic) combined with comorbidities (e.g., hypermobility syndromes) and the functional impairment has been compared to that seen in chronic obstructive pulmonary disease or congestive heart failure [25].

In this case the diagnostic work-up of PoTS led to the diagnosis of autoimmune-mediated neuropathic PoTS as a crossroad of PoTS, SFN and autoimmunity. The characterisation of autoantibodies against adrenergic and muscarinic receptors is nowadays performed in specialized laboratories with the use of life-based bioassays on cardiomyocytes confirming the functional role of these autoantibodies [34]. The relevance of these antibodies for disease initiation and activity is still under investigation. However, in our case the titers of these antibodies improved during therapy in accordance with the stabilization and improvement of clinical symptoms. Consequently, the existence of these antibodies implies that autoantibodies could be more important than cytotoxic T-cell attack since the antibody titers were reduced after treatment as previously reported for other diseases [35].

This aspect has important implications for medical care, given the widespread availability and proven efficacy of immunotherapies for autoimmune neuropathies. IVIg is increasingly prescribed off-label for autoimmune PoTS and SFN following dosing parameters established in chronic inflammatory demyelinating polyneuropathy (CIDP) trials [30,36,37].

However, supplies of IVIg are limited and administration can be challenging. IVIg often causes infusion reactions and rarely causes serious adverse events. Thus, adapted dosing schemes and premedication are suggested in autonomic neuropathies [11,21,37]. In light of the novel subcutaneous route of application for immunoglobulin for CIDP [19], we show that SCIg is an attractive option for autoimmune neuropathic PoTS, due to the improved side-effect profile. Tolerating the medication is probably mostly relevant for patients with autonomic neuropathies, as dysautonomia patients are likely to be more vulnerable to infusion-associated reactions using IVIg standard protocols [21,37].

Concluding, surely this non-controlled case presentation does not provide adequate data for the immunomodulatory effect of IVIg/SCIg in autoimmune neuropathic SFN. A placebo effect cannot be ruled out. However, since the first randomized, double-blind, placebo-controlled, clinical trials of IVIg for idiopathic SFN and autoimmune neuropathic POTS have begun recruitment [18,38], further studies are urgently needed to investigate tolerability and efficacy of SCIg as treatment option in treatment refractory autonomic SFN and neuropathic POTS.

## Funding

The authors have not declared a specific grant for this research from any funding agency in the public, commercial or not-for-profit sectors.

## Author contributions

All authors have read and approved the manuscript. Kalliopi Pitarokoili: First idea, patient treatment, acquisition, analysis and interpretation of data, drafting and manuscript revision, study supervision. Andrea Maier: first diagnosis, patient treatment, acquisition, analysis and interpretation of data, drafting revising the manuscript. Elena de Moya: drafting/revising the manuscript for content, patients’ insights. Katrin Hahn: first treatment with IVIg, patient treatment, drafting/revising the manuscript for content. Gerd Wallukat: antibody studies, drafting/revising the manuscript for content. Jeremias Motte: patient treatment, acquisition, analysis and interpretation of data, drafting/revising the manuscript for content. Thomas Grüter: acquisition, analysis and interpretation of data, drafting/revising the manuscript for content. Athanasopoulos Diamantis: patient treatment, acquisition, analysis and interpretation of data, drafting/revising the manuscript for content. Anna Lena Fisse: acquisition, analysis and interpretation of data, drafting/revising the manuscript for content. Ralf Gold: Critical comments during data collection, drafting and manuscript revision.

## Financial disclosures

Kalliopi Pitarokoili: serves on scientific advisory boards for Celgene and German POTS und andere Dysautonomien e.V.; has received speaker honoraria and travels grants from 10.13039/100006314Biogen Idec, 10.13039/100006259Teva Pharmaceutical Industries Ltd., 10.13039/501100006010Bayer Schering Pharma, 10.13039/100006436Celgene, 10.13039/100008322CSL Behring, 10.13039/501100016387Grifols and 10.13039/100004336Novartis; her research is funded by 10.13039/501100007316Klaus Tschira Foundation and 10.13039/501100008835Ruhr-University, Bochum (FoRUM-program); none related to this manuscript.

Andrea Maier: serves on scientific advisory board for German POTS und andere Dysautonomien e.V. Funding for travel or speaker honoraria: German society for ME/CFS, expense allowance for lecture German Ehlers Danlos Initiative, expense allowance for lecture CentoGene AG, travel allowance study meeting Shire, Travel expenses congress on M. Fabry, Research Support, travel expenses for lecture German POTS und andere Dysautonomien e.V.

Foundations and Societies: Parkinson Fonds International Research Grant, StandingUptoPOTS Research Grant, Financial support for a register on autonomic neuropathies from 10.13039/100007723Takeda.

Elena C. de Moya Rubio is the co-founder and president of the German Patient NGO POTS und andere Dysautonomien e.V., and has nothing to disclose.

Katrin Hahn: serves on scientific advisory boards for Akcea, Pfizer and Alnylam; has received speaker honoraria from Akcea, Pfizer and Alnylam; and receives research support from Akcea, 10.13039/100004319Pfizer and 10.13039/100006400Alnylam, none related to this manuscript.

Gerd Wallukat: is “Director for Research and Development” at the Berlin Cures GmbH, the AAB estimation was performed within a PoTS project and was free of charge, he has nothing to disclose.

Jeremias Motte: received travel grants from 10.13039/100006314Biogen idec, 10.13039/100004336Novartis AG, 10.13039/100006259Teva and 10.13039/501100003769Eisai GmbH, his research is funded by 10.13039/501100007316Klaus Tschira Foundation and 10.13039/501100008835Ruhr-University, Bochum (FoRUM-program); none related to this work.

Thomas Grüter: received travel reimbursement from Sanofi Genzyme and Biogen Idec, none related to this manuscript.

Diamantis Athanasopoulos has nothing to disclose.

Anna Lena Fisse: received research funding by Georgius Agricola Stiftung Ruhr and 10.13039/501100008835Ruhr-University, Bochum (FoRUM-program), received honoraria and travel grants from 10.13039/100004336Novartis AG, 10.13039/100004339Sanofi and 10.13039/501100003769Eisai GmbH, none related to this work. Owns shares of Fresenius SE & Co., Gilead Sciences, Medtronic PLC and Novartis AG.

Ralf Gold: serves on scientific advisory boards for Teva Pharmaceutical Industries Ltd., Biogen Idec, Bayer Schering Pharma, and Novartis; has received speaker honoraria from Biogen Idec, Teva Pharmaceutical Industries Ltd., Bayer Schering Pharma, and Novartis; serves as editor for Therapeutic Advances in Neurological Diseases and on the editorial boards of Experimental Neurology and the Journal of Neuroimmunology; and receives research support from 10.13039/100006259Teva Pharmaceutical Industries Ltd., 10.13039/100006314Biogen Idec, 10.13039/501100006010Bayer Schering Pharma, 10.13039/100004329Genzyme, Merck Serono, and 10.13039/100004336Novartis, none related to this manuscript.

## Ethical standards

All procedures performed in studies involving human participants were in accordance with the ethical standard of the institutional and/or national research committee and with the 1964 Helsinki declaration and its later amendment or comparable ethical standards.

## Patient and public involvement

The patient gave spoken and written consent for publication and participation to the study and is co-author of this article, including needs and insights, such as quality of life following the Patient and Public Partnership (PPP) principles and establishing the patient as an expert in her rare disease (RD), and implementing the newest research standards for RD [39].

## Data sharing statement

Data collected from this study are available by emailing Kalliopi. Pitarokoili@ruhr-uni-bochum.de.
